# Supplementary material for: How social media influences the self-image and body image among female and male adolescents
Source: PLoS One. 2025 Nov 26;20(11):e0334657. doi: 10.1371/journal.pone.0334657 (PMC12654893; doi:10.1371/journal.pone.0334657)
Supplement: S1 File — (DOCX) [file pone.0334657.s001.docx]

| Theme 1 | Theme 1.1 | Theme 2 | Theme 2.2 |
| --- | --- | --- | --- |
| Elizabeth (female, aged 15–16 years): If a person posts a picture on social media that you think looks nice, and there are only positive comments below the photo, then that's where you set your standards.” | Sarah (female, 17-year-old): “Social media are something I use to get in a better mood, and I’ve noticed that I can achieve a better self-image due to social media.” | Markus (male, aged 17-18 years): “(…) like body-image pressure, for instance. I got tired of watching it [content related to body image], so I just deleted it (TikTok).” | Maria (female, aged 18–19 years): “To see that people look more like you (…), that can only be positive.” |
| Peder (male, aged 16–17 years): “It makes you feel like you’re not good enough if you don’t have that perfect body. You feel like you’re supposed to have a proper six-pack, huge muscles, and be over 180 [cm tall],” | Suzanne (female, aged 17–18 years: "Snapchat is more unpretentious in a way (…). On Instagram, things are supposed to look nice and decent.” | Hannah (female, aged 16–17 years): It influences your subconsciousness. (…) I don't care much if people post a bikini picture; I scroll past it. But your subconsciousness can pick up on it in a way, and later, you start thinking, 'Oh, I need to be thinner.’ | Olivia (female, aged 17–18 years): “You are supposed to like your body, but why should we focus this much on our bodies? (…) People say, 'It's who you are that's important,' but then they post half nudes telling others to love their body.” |
| Olaf (male, age 16–17 years): “Girls focus on things that are not possible to fix, like height and physique | Hannah (female, aged 16–17 years): “If you follow a certain [social media] account, then similar accounts appear. So it [body-image pressure] is just getting stronger and stronger,” |  |  |
| Adrian (male, aged 16–17 years): "Men have feelings, you know,” | Hayley (female, aged 15–16 years): It [body-image pressure] is mainly on Instagram, but there is a lot on TikTok too. On Instagram, I have chosen to follow girls or models who look really, really good. But on TikTok, these things show up. |  |  |
| Tom (male, aged 15–16 years): “I don’t think boys feel the same pressure as girls. |  |  |  |
| Simon (male, aged 15–16 years): "It's not like they [males] feel that they must be perfect or have this or that body just because someone has posted something on Instagram. Boys simp a little less.” |  |  |  |
| Peter (male, 18-year-old): "It [social media] can either drastically improve my mood or bring it down, depending on what I'm seeing. In my opinion, my self-image is not affected by social media since I'm already confident that I am good-looking.” |  |  |  |
| Tomas (male, 16-year-old):  Social media haven't influenced me; this is because I am satisfied with my body and how I look. It's also because the pictures others post are unrealistic and usually created with a specific posture or lighting to make you look better. |  |  |  |
