# Supplementary material for: How social media influences the self-image and body image among female and male adolescents
Source: PLoS One. 2025 Nov 26;20(11):e0334657. doi: 10.1371/journal.pone.0334657 (PMC12654893; doi:10.1371/journal.pone.0334657)
Supplement: Attachment — Submitted filename: Response to Reviewers.docx [file pone.0334657.s004.docx]

Dear editor and reviewers.

Dear Dr. Annesha Sil

Thank you for considering our manuscript “How social media influences the self-image and body image among female and male adolescents*”* for publication in PLOS ONE.

Thank you for your valuable and thorough feedback on our manuscript. We have included responses to all comments below, highlighted in track changes within the manuscript, and included the line number below where changes can be found. We have reworked the aims by incorporating the two methods, reworking the analysis, and triangulating the results from the focus groups and survey. Further, we have refined the results and removed some of the statements from the manuscript to keep focus on the main findings. Additional statements have been included as supporting information, along with the anonymous survey data. We hope our responses are satisfactory and that the manuscript meets the standards for publishing in PLOS ONE.

On behalf of the authors,

Yours sincerely

**Merete Kolberg Tennfjord**

Associate professor

Physiotherapist

Editor comments to the author

3. In this instance it seems there may be acceptable restrictions in place that prevent the public sharing of your minimal data. However, in line with our goal of ensuring long-term data availability to all interested researchers, PLOS’ Data Policy states that authors cannot be the sole named individuals responsible for ensuring data access (<http://journals.plos.org/plosone/s/data-availability#loc-acceptable-data-sharing-methods>).

Data requests to a non-author institutional point of contact, such as a data access or ethics committee, helps guarantee long term stability and availability of data. Providing interested researchers with a durable point of contact ensures data will be accessible even if an author changes email addresses, institutions, or becomes unavailable to answer requests.

Before we proceed with your manuscript, please also provide non-author contact information (phone/email/hyperlink) for a data access committee, ethics committee, or other institutional body to which data requests may be sent. If no institutional body is available to respond to requests for your minimal data, please consider if there any institutional representatives who did not collaborate in the study, and are not listed as authors on the manuscript, who would be able to hold the data and respond to external requests for data access? If so, please provide their contact information (i.e., email address). Please also provide details on how you will ensure persistent or long-term data storage and availability. **Response**: Due to participant privacy, the qualitative data material from the focus groups will not be shared. The online data is anonymous and will be shared as a supplementary file alongside this revision. We have updated this statement in the online submission system.

4. We note that you have included the phrase “data not shown” in your manuscript. Unfortunately, this does not meet our data sharing requirements. PLOS does not permit references to inaccessible data. We require that authors provide all relevant data within the paper, Supporting Information files, or in an acceptable, public repository. Please add a citation to support this phrase or upload the data that corresponds with these findings to a stable repository (such as Figshare or Dryad) and provide and URLs, DOIs, or accession numbers that may be used to access these data. Or, if the data are not a core part of the research being presented in your study, we ask that you remove the phrase that refers to these data. **Response**: Thank-you for this comment. We have removed the data related to age as these are not part of the research question.

5. Review Comments to the Author

Please use the space provided to explain your answers to the questions above. You may also include additional comments for the author, including concerns about dual publication, research ethics, or publication ethics. (Please upload your review as an attachment if it exceeds 20,000 characters)

Reviewer #1: 1. Is the manuscript technically sound, and do the data support the conclusions?

The manuscript is overall good. It presents a compelling exploration of an important topic in adolescent psychology, focusing on both females and males’ ideas and perceptions related to social media. The authors attempt to integrate various sides of the story when it comes to body image and self-image and try to touch on both. The results are interesting but maybe this section could benefit from further refinement in terms of organization and clarity. The research questions and data do support the conclusions in this manuscript.
**Reply**: Thank-you for your comment. We have revised the analysis and organized the results into two main themes and two sub-themes covering both the survey and the focus-group interviews. We have further removed some statements from the manuscript and included them as supplementary information organized under the different themes.

*2. Has the statistical analysis been performed appropriately and rigorously?

The statistical analysis of this manuscript appears to be appropriately and rigorously performed. The authors showed attention to detail and provided a strong foundation for the analysis. **Response**: thank-you for this comment.

*3. Have the authors made all data underlying the findings in their manuscript fully available?

No, the authors declared that there are some restrictions for data availability as well as that they have no consent to share data sampled from the interviews undertaken. **Reply**: Due to participant privacy, the qualitative data material from the focus groups will not be shared. The online data is anonymous and will be shared as a supplementary file alongside this revision. We have updated this statement in the online submission system.

*4. Is the manuscript presented in an intelligible fashion and written in standard English?

Overall, the manuscript presented in an intelligible fashion and written in standard English. There are a few minor grammar mistakes that are not that much important, but should be addressed with a brief review. **Reply**: We have reviewed the manuscript for grammatical errors and hope this work is satisfying.

*5. Review Comments to the Author


Notes:
- Abstract: missing the background of the topic. **Response**: Thank-you for your comment. We have included relevant background information for the abstract.

- Introduction: overall comprehensive and provides a well-established overview of the topic. **Response**: Thank-you for your positive feedback.

- Line 49-50: what is that sociocultural influence? Maybe give examples for the sentence is a bit ambiguous. **Response**: Thank-you for the comment. We have included some additional information for clarity and included the reference of Thompson et al 1999 that explains this relationship more in detail (line 110). We have also included some clarification related to personalization on social media and included a reference from our research group showing adolescents limited awareness of social media personalization (line 117).

- Limitation for the methodology section: sampling bias as the adolescents were recruited from one municipality. **Response**: thank-you for your comment. We agree that this information should be included, and we have included information about sampling bias (line 1094) and the cross-sectional design (line 1106) in the limitations. Related to the social desirability bias, we discussed this in the results section and further in the discussion, and also included a comment in the limitations section on line 1050.

- I do not know about using the adolescents first names in the results? Is that okay? **Response**: The adolescents from the interviews were given fictitious names to ensure anonymity. This information was included in line 328.

- The themes identified are very interesting, however they are presented in an excessively lengthy manner and could be rephrased to be shorter and more concise. **Response**: Thank-you for this comment. We have reworked the themes into two main themes and two sub-themes, triangulating the results from both the survey and the focus-groups.

- Discussion: well-structured and comprehensive discussion – compares and backs up the results of the study with previous literature, included both males and females. Also, there were different theory integration when explaining how social media effects self-image and body image, such as the social comparison theory. **Response**: Thank-you for your positive feedback. We have added a few points to the discussion following the reorganization of the results.

- The implications for clinical practice section in the discussion was important to add as it enhances the comprehensiveness and relevance of the study’s findings, giving an idea for targeted interventions and support services to better understand adolescents’ experiences with regards to social media and body image. **Response**: Thank-you for your positive feedback.

Reviewer #2: Thank you for the opportunity to review this manuscript.

The subject of self-image and its relations to social media is certainly relevant and there are many nuances to be explored within it. However, I think the manuscript has methodological issues that need to be addressed before it can be published.

1. Firstly, I lacks focus, trying to cover too many objectives and using different types of data that are not triangulated at the analytical and interpretative stages. The paper has currently four objetives: to investigate social media influences on adolescents` body-image; how adolescents view their body image in relation to their self image with emphasis on gender differences; to investigate adolescents’ social media practices; and their experiences with body positivity. My impression is that the thematic analysis was not fully completed, not reaching the stage when sub-themes are systematized into themes.
**Response**: Thank-you for your valuable comment. We agree that the objectives are too extensive, and we have rephrased accordingly (line 216). A detailed explanation of the re-organisation of the methods, analysis and results, triangulating the two datasets are discussed below under point 3.

2. Secondly, the data collection procedures have limitations that need to be considered in your analysis. The main one refers to the questions that were asked during FG, which induce participants to confirm that SM influences them and their body-image (“How does what you see on social media influence you?”) and some are not open questions (“Do you believe that social media contribute to body appearance pressure?”). The questions regarding SM use do not seem to have been fully analyzed or at least the results are not reported. Two other limitations are: 1. the fact that the FG were done in schools, an institutional scenario where adolescents will not necessarily feel comfortable to talk about themselves and their feelings, which may have contributed to their discourse being more influenced by social desirability; 2. only female researchers conducted the FG, which may have had an important impact on how boys expressed themselves (or refused to).

**Reply**: Thank-you for your comments. Related to the question: “Do you believe that social media contribute to body appearance pressure?”, this question gave us a more confirmatory response as the issues of body image pressure had already been explored through other questions. We have included some additional information on line 314. From this question, the adolescents also responded more objectively in relation to social media platforms as covered under theme 1.1. We have included more reflections on the questions asked in the limitations section.

Related to the question: “How does what you see on social media influence you?”, we have rephrased the question as it was posed: “In what way would you say that social media influence you?”. We acknowledge that this question may be leading in a way that the adolescents did confirm that SoMe influenced them. Based on the literature, a predominantly negative influence of SoMe has been published. Thus, we were interested in a more nuanced exploration covering negative and positive feelings towards this influence. We have included some more reflections in the limitations section related to the questions asked in both the survey and the focus-groups. However, we believe we have gained insight into various feelings and experiences of both self-image and body image among both males and females participating.

The questions “Is there any common theme on the social media accounts you follow?”, “Which social media accounts do you follow?”, and “What do you look at while on social media?” were included for the overall project and included in the interview but these questions gave no relevant data to the objectives of this study. We have therefore removed these questions from the current study.

Regarding the school as location, this was done for convenience due to the students taking time off from classes to join the project. Further, sampling students outside of the school environment could have resulted in groups of students not being familiar with each other, reducing the feeling of trust and safety among each other. We acknowledge that the school environment may for some be difficult and uncomfortable, but by creating various environments (same-gender and mixed-gender groups) we aimed to explore the social contexts within these different groups. In line 267 and 291 we have elaborated on the planning of the focus groups and included some information about the choices made for location.

Regarding the inclusion of a female interviewer, we agree that including a male interviewer could have made the male adolescents speaking more openly about social media influence. We have included this information in the limitations section.

3. When we get to the analysis, the results are presented in the form of two separated research questions, reflecting the lack ofdata triangulation. In addition, the first research question contains three questions in ti: "How do social media influence adolescents’ body-image, what are their social media practices and experiences with body positivity?” My suggestion would be to reframe the aims into one that integrates the others and then rework your data analysis, either triangulating the results from FG and questionnaires or focusing in further analyzing one of them (probably the FG), making sure you accomplish all the stages of the thematic analysis.
**Reply**: We have rephrased the aims into one that incorporates both datasets. We have further included a separate section explaining the triangulation of the analysis for the survey and the focus-groups (line 424). The results are thus presented including both data in an integrated manner covering adolescents’ feelings of their self-images and body images. We have refined the limitations section accordingly.

One last observation: the title needs to be revised. Although the current one is catchy, it does not reflect the main message of your findings. **Reply**: Thank-you for your comment. We have changed the title accordingly.
