# Supplementary material for: How social media influences the self-image and body image among female and male adolescents
Source: PLoS One. 2025 Nov 26;20(11):e0334657. doi: 10.1371/journal.pone.0334657 (PMC12654893; doi:10.1371/journal.pone.0334657)
Supplement: Attachment — Submitted filename: response_to_reviewers_auresp_2.docx [file pone.0334657.s005.docx]

Dear editor and reviewers.

Dear Dr. Emily Lowthian

Thank you for the opportunity to revise our manuscript, “How social media influences the self-image and body image among female and male adolescents,” for possible publication in **PLOS ONE**.

We have carefully considered the insightful comments from both reviewers and have revised the manuscript accordingly. A detailed, point-by-point response to each comment is provided below. We have also uploaded a revised version of the manuscript with all changes clearly highlighted.

We sincerely appreciate the reviewers’ constructive feedback, which has helped us improve the clarity and rigor of our work. We hope that our revisions meet the journal’s requirements and address the reviewers’ concerns satisfactorily.

Thank you for your consideration of our revised manuscript. We look forward to your feedback.

Sincerely,
Merete Kolberg Tennfjord

Kristiania University of Applied Sciences
Corresponding Author Email: meretekolberg.tennfjord@kristiania.no

Reviewers' comments:

Reviewer's Responses to Questions

**Comments to the Author**

1. If the authors have adequately addressed your comments raised in a previous round of review and you feel that this manuscript is now acceptable for publication, you may indicate that here to bypass the “Comments to the Author” section, enter your conflict of interest statement in the “Confidential to Editor” section, and submit your "Accept" recommendation.

Reviewer #2: All comments have been addressed

Reviewer #3: (No Response)

**Response**: No addition comment

2. Is the manuscript technically sound, and do the data support the conclusions?

The manuscript must describe a technically sound piece of scientific research with data that supports the conclusions. Experiments must have been conducted rigorously, with appropriate controls, replication, and sample sizes. The conclusions must be drawn appropriately based on the data presented.

Reviewer #2: Yes

Reviewer #3: Yes

**Response**: no additional comment

3. Has the statistical analysis been performed appropriately and rigorously?

Reviewer #2: I Don't Know

Reviewer #3: Yes

**Response**: no additional comment

4. Have the authors made all data underlying the findings in their manuscript fully available?

The [PLOS Data policy](http://www.plosone.org/static/policies.action#sharing) requires authors to make all data underlying the findings described in their manuscript fully available without restriction, with rare exception (please refer to the Data Availability Statement in the manuscript PDF file). The data should be provided as part of the manuscript or its supporting information, or deposited to a public repository. For example, in addition to summary statistics, the data points behind means, medians and variance measures should be available. If there are restrictions on publicly sharing data—e.g. participant privacy or use of data from a third party—those must be specified.

Reviewer #2: No

Reviewer #3: Yes

**Response**: comment provided in last review.

5. Is the manuscript presented in an intelligible fashion and written in standard English?

PLOS ONE does not copyedit accepted manuscripts, so the language in submitted articles must be clear, correct, and unambiguous. Any typographical or grammatical errors should be corrected at revision, so please note any specific errors here.

Reviewer #2: Yes

Reviewer #3: Yes

**Response**: no additional comment

6. Review Comments to the Author

Please use the space provided to explain your answers to the questions above. You may also include additional comments for the author, including concerns about dual publication, research ethics, or publication ethics. (Please upload your review as an attachment if it exceeds 20,000 characters)

Reviewer #2: Thank you for addressing the concerns I raised in the previous review. This version of the paper is clearer, but some sections still require further attention, as detailed below:

- The first sentence of the abstract ("The primary driver for social comparison is social media, where upward comparisons seem to harm young people's self-images and body images") is not fully correct and not in accordance with the first sentence of the introduction. I suggest replacing it with the latter.

**Response**: Thank-you for your comment, we have rewritten the abstract accordingly.

- Several sentences could benefit from a more scientific tone, particularly by introducing some nuance or uncertainty. For instance, consider revising the use of words like "obvious" and "discovered" in the following examples:
- "It was obvious from our results that most adolescents viewed the impact of social media as negative."
- "Triangulating these data, we discovered the overall negative influence of social media on their self-image and body image.”

**Response**: Thank-you for your comment, we have rewritten the above sentences accordingly.

- In the triangulation topic, the following information that should be in the results, not methods : ”It was obvious from our results that most adolescents viewed the impact of social media as negative. Further, the descriptions of adolescents' self-images were related to appearance and unattainable body ideals. Thus, the findings from the survey aligned with the results of theme one, as identified from the focus group analysis. For theme two, the survey participants' positive responses aligned with the focus groups' responses, with some adolescents actively pursuing positive content on social media to improve their self-image and body image. However, a more in-depth understanding of this behavior, particularly among male participants, emerged from the focus groups. Lastly, experiences of body positivity were evident only from the focus groups and were not supported by survey data.”

**Response**: Moved to results section.

- The categorization process in the survey is unclear. The sentence "Dividing the survey statements into categories, it was indicated that 55 respondents (44.4%) reported that social media had a negative influence on their self-image, compared with 20 (16.1%) who provided positive reports and 49 (39.5%) who reported mixed or neutral feelings (Table 2)" needs further clarification. Also, was the difference between negative and mixed-feeling responses statistically significant?

**Response**: The categorization of the survey statements was done qualitatively. First by each author, before a joint evaluation and revision was done. We have added more information about this process in the methods on page 14 and in the limitations. And rewritten the paragraph in the results section on page 14 and 15. There was no difference between gender on the negative or mixed feelings. We have added this sentence on page 16.

- The authors affirm that "Most of their described self-images were related to appearance and unattainable body ideals, giving the impression that body image plays a significant role in adolescents' self-images, more so for females than males. Pia, aged 16, said, "My self-image is influenced by ideals I know I cannot fulfill.” The quote does not explicitly refer to body image, and could be about other aspects of self-image, such as routine, housing, or leisure, which have also been shown to be influenced by social media in the literature.

**Response**: We agree that self-image may contain various aspects, not simply body image, and that this quote also may imply different things. We have therefore nuanced the quote from Pia 16 years, and moved the section about self-image and body image so that it matches the quote from Mona 20 years. We have further expanded on this as a limitation in line with your comment below.

- In several parts of the results and discussion, the concepts of body image and self-image seem to be conflated. For instance, in the conclusions, you state: "in the survey, we strived to capture the adolescents’ reflections on their self-image and how body image could relate. However, some may have been unfamiliar with the term self-image, which could reflect a relatively narrow comprehension of participants` self-image and, thus, their reflections on body”. How can you be certain that respondents were unfamiliar with the term "self-image" in an online survey?

**Response**: We have rewritten parts of the results and discussion, trying to avoid the conflation of these terms and thus nuancing the findings from the survey. We have also rewritten part of the limitation section bringing in how self-images may also be related to personal characteristics and attributes.

- In the following sentence, there is no contradiction, but addition: Some participants mentioned in the survey that Instagram influenced their self-image. However, in the interviews, when asked whether social media contributes to body-image pressure, the participants suggested that this is most prevalent on Instagram, followed by Snapchat and TikTok

**Response**: We have corrected the wording accordingly.

- In the opening sentence of the discussion ("This study has provided insight into the experiences of females and males with social media and how social media influences their self-image and body image”)- please add where (Norway)

**Response**: Norway is added in the first sentence

- In the discussion, you evoke results regarding health that do not seem to have been presented in the manuscript and that exceed your objectives: "Goodyear et al. [35] found that health-related information, such as on diet and exercise, 770 can positively affect the health-related behaviors of young males and females. However, 24% of the younger adolescents in the latter study reported that the health-related content they see on social media had a negative impact on their health-related behaviors.”

**Response**: we have rewritten the sentence, referring to the study by Goodyear for these numbers.

- I do not see data supporting the age variation you refer to in this sentence:"Although these are positive findings for VSCO, this platform was only mentioned twice, both times by female participants, suggesting that the impacts of less approval-oriented platforms can vary with age and gender”.

**Response**: We have removed age from this sentence.

- Considering that your data provides more substantial evidence of social media’s effects on females, I was surprised by the first conclusion: "greater attention must be paid to the influences of social media on males." Having read both versions of the manuscript, and noting that the first title focused on males, it seems that there may be a particular interest in studying this group, which could introduce a bias in your interpretation.

**Response**: Thank-you for your comment. We strived to capture both females and males experience of social media influence and were particular interested in any discrepancies between them. Indeed, our sample has more females, in both the survey and interviews, which we have discussed as a limitation in our manuscript. Further, during the interviews we had an open and curious approach to capture all the participants voices. In the analysis, we strived to capture differences and similarities between different age groups and gender, keeping the open approach to the data. Please see page 11 in the methods for this information. We believe these precautions have reduced the influence of bias in our interpretation. We have therefore rewritten the implications section to emphasize that our study brings evidence of both genders, but that our study in addition brings evidence for social media impact on young males.

Reviewer #3: I commend the authors on their examination of the impact of social media on adolescents' self-image and body image. The combination of focus group interviews (qualitative) and survey data (quantitative) strengthens the study by providing in-depth insights. Many studies on body image focus on female experiences, but this paper includes both males and females, offering an important contribution to the literature on gender differences in how adolescents experience social media influence. While the study provides valuable findings, some areas require improvement:

**Response**: Thank-you for your positive feedback

Abstract
1. Additional demographic details such as the percentage of female participants in both studies and the mean age of participants should be included for better contextualization of the sample.
2. Additionally, a minor grammatical correction is needed in line 36: “Triangulating these data, discovered the overall negative influence of social media on their self-image and body image.’. It can be corrected to, “Triangulating these data revealed the overall negative influence of social media on their self-image and body image.”.

**Response**: We have added the demographic details in the abstract and rewritten the abstract sentence also nuancing the findings

Introduction section
3. In lines 49-53, “Social media is considered the most pervasive and persuasive of these drivers. This is due to applications being largely visual platforms, which ceaselessly expose young people to a wide assortment of idealized images that reflect appearance and performance ideals as well as other personal characteristics.”, this claim needs a citation to support it, as it makes a broad assertion about social media’s role in sociocultural influence. For example, the authors can use data from Statista.
Dixon, S. (2023). Social media—Statistics & Facts. Statista. <https://www.statista.com/topics/1164/social-networks/>

**Response**: we have added the reference as recommended

4. Additionally, there is no dedicated paragraph on how social media influences self-image and body image. The authors should include a paragraph addressing current literature on social media’s associations with self-image and body image.
Given this, it should be noted that the authors should present a balanced viewpoint of both negative and null associations of social media on body image. For example, recent studies have found both negative (e.g., Fardouly & Vartanian, 2016) and null associations between objective social media screen time and body image dissatisfaction (Goh et al., 2025). There should be more literature review on both positive and negative findings of the associations.
Fardouly, J., & Vartanian, L. R. (2016). Social Media and Body Image Concerns: Current Research and Future Directions. Current Opinion in Psychology, 9, 1–5. https://doi.org/10.1016/j.copsyc.2015.09.005
Goh, A. Y. H., Hartanto, A., Kasturiratna, K. T. A. S., & Majeed, N. M. (2025). No Consistent Evidence for Between- and Within-Person Associations Between Objective Social Media Screen Time and Body Image Dissatisfaction: Insights From a Daily Diary Study. Social Media + Society, 11(1), 20563051251313855. https://doi.org/10.1177/20563051251313855

**Response**: Thank-you for your insightful comment. We have included some more information about social media and the balanced view of both negative and null associations on body image and self image.

Methods; Focus Group Interviews and The Survey
5. In line 181 and line 266, there is clarification needed with regard to how screen time was measured (“The mean time spent on social media by the 48 participating adolescents was 4.8 hours per day, and was the same for both genders.” and “The time spent on social media was 4.48±2.30 hours overall, with females spending 4.74±2.24 hours and males spending 4.16±2.34 hours.”). Is this self-reported screen time, or was it objectively measured through an app or tracking software?
If self-reported, the authors should acknowledge the potential bias in recall accuracy.
Wilcockson, T. D. W., Ellis, D. A., & Shaw, H. (2018). Determining Typical Smartphone Usage: What Data Do We Need? Cyberpsychology, Behavior, and Social Networking, 21(6), 395–398. https://doi.org/10.1089/cyber.2017.0652

**Response**: the reported screentime was self-reported which we have added to the methods. We have also added the reference in the limitation section about the potential bias in recall accuracy.

6. The authors should also list the 15 questionnaire items or provide more specificity regarding the aspects of social media measured, line 252, “The survey contained demographic questions that included gender, 15 questionnaire items on different aspects of social media.”.

**Response**: We have specified the questionnaire items in the methods section. The responses to these questions will appear in a separate publication.

7. Given that the survey had a low response rate (39.62%), it raises concerns about self-selection bias. The Discussion section should address this and discuss how it affects generalizability.

**Response**: Yes, we agree that the response rate may affect generalizability. We have thus included this as a limitation discussing the self-selection bias in the limitations of the study.

8. The authors categorized open-ended responses into positive, negative, and neutral, but do not specify how intercoder reliability was measured. Given this, agreement rates between authors, if applicable, can be reported to ensure transparency.

**Response**: The categorization of the survey statements was done qualitatively. First by each author, before a joint evaluation and revision was done. We have added more information about this process in the methods on page 14 and in the limitations section. And rewritten the paragraph in the results section on page 15.

9. Was a thematic map used to organize qualitative findings? If so, including it as a figure would improve clarity.

**Response**: We have created a figure showing the 6 steps from Braun & Clarke with illustrations from our data material (Figure 1). Importantly, the final themes as included in this manuscript was further refined after step 5 due to data triangulation. We have also created a second figure showing the processes in a thematic map.

Results and Discussion section
10. The results suggest that participants felt Instagram influenced their self-image the most, followed by Snapchat and TikTok (lines 358-361, “However, in the interviews, when asked whether social media contributes to body-image pressure, the participants suggested that this is most prevalent on Instagram, followed by Snapchat and TikTok.”). The authors should clarify whether this ranking is due to the nature of these platforms or simply because they are the most used applications.
If platform usage frequency explains this pattern, it should be highlighted in the discussion section as a limitation.

**Response**: According to Statistics Norway, Instagram is used by 81% of adolescents between 16 and 19 years, followed by Snapchat which is used by 93% and TikTok with 83%. We agree that it might be the frequency of usage that may explain the influence and not necessarily the nature of this platform. We have included this information in the discussion section.

This manuscript offers valuable insights into the influence of social media on adolescents' self-image and body image, particularly in relation to gender differences. However, several areas require improvement. The introduction would benefit from a stronger theoretical foundation, particularly through the inclusion of relevant literature on social media’s associations with self-image and body image. In the methods section, clearer explanations are needed regarding screen time measurement, questionnaire items, and the potential impact of the survey’s low response rate on generalizability. Additionally, clarifications in data reporting—such as resolving inconsistencies in screen time estimates and providing transparency on intercoder reliability—are necessary to meet academic standards. Addressing these revisions will significantly enhance the manuscript’s clarity, methodological rigor, and overall impact.

**Response**: Thank-you for your valuable feedback. We hope we have clarified your concerns in the revised manuscript. We have addressed your comments above.
